# Epidemiology and risk factors for phantom limb pain

**DOI:** 10.3389/fpain.2024.1425544

**Published:** 2024-08-21

**Authors:** Shoji Ishigami, Carol Boctor

**Affiliations:** ^1^ Department of Physical Medicine and Rehabilitation, School of Medicine, West Virginia University, Morgantown, MV, United States; ^2^ School of Medicine, West Virginia University, Morgantown, MV, United States

**Keywords:** phantom limb pain (PLP), risk factors, amputation, epidemiology, phantom pain

## Abstract

Approximately 356 million limb amputations are performed globally every year. In 2005, the prevalence of limb loss in the United States was 1.6 million people; and it is estimated to increase to 3.6 million by 2050. Many post-amputation patients experience chronically altered sensations and pain associated with the amputation, such as phantom limb pain. The risk factors for phantom limb pain are widely debated in the literature due to the heterogeneity of the population being studied. This review will highlight both the non-operative and operative risk factors for phantom limb pain.

## Epidemiology

Approximately 356 million limb amputations are performed globally every year (1). In the United States, the prevalence of limb loss was 1.6 million people in the year 2005; and it is estimated to increase to 3.6 million people by the year 2050 (2). There are about 185,000 amputations performed annually in the U.S. due to vascular disease (82%), trauma (16.4%), cancer (0.9%), and congenital anomalies (0.8%) (3, 4). Amputations are also more common among older individuals, men, and non-white person/persons/people. The total projected lifetime healthcare costs after lower limb amputation were staggering at $509,275 per patient. In addition to accruing that cost, 42% of those patients reported being unable to work for 7 years following their lower limb amputation, further exacerbating the financial and psychological burden (5, 6).

Most of the post-amputee patients experience chronically altered sensations and pain associated with the amputation. Aside from the expected post-surgical pain, these patients can also experience phantom limb pain (PLP), sustained residual stump pain or residual limb pain (RLP), phantom limb sensation (PLS), and telescoping phenomena. In 2020, Stankevicius et al. showed that up to 82% of post-amputees developed PLP within one year from their amputation; and that the lifetime prevalence for PLP and PLS remained equally high (76%–87%, and 87% respectively) (7). They also reported that approximately 25% of amputees experienced telescoping. Another study determined that the prevalence of PLP is estimated to be as low as 64% (8).

## Risk factors

This discrepancy in PLP prevalence led to challenges in mitigating the complications of altered sensations. This is largely in part due to the heterogeneity of the population being studied and the historical tendency of physicians to focus more on the non-operative risk factors. However, the PLP risk factors can be divided into 3 categories: pre-operative, peri-operative, and post-operative stages of amputation.

Pre-operative risk factors positively correlated with developing PLP are divided into nonmodifiable and modifiable factors (8–17). Nonmodifiable factors may include age, sex, and race/ethnicity. Modifiable factors can include lack of socioeconomic support, the severity of chronic conditions (ex. diabetes mellitus), preoperative pain levels, absence of pre-procedure counseling, absence of congenital limb loss, and preexisting psychiatric disorders such as anxiety, depression, or tendencies towards catastrophizing. Over the last 8 years, studies displayed some consensus on the two main pre-operative risk factors for PLP (18–20). The first main risk factor is older age. The second is the efficacy of previous pain treatments suggestive of reduced intrinsic capacity for cortical reorganization after amputation.

The risk factors for PLP in the perioperative stage are mainly the surgical techniques utilized during amputation. Emerging evidence suggests that advanced surgical techniques seem to reduce the onset of PLP (21–26). A commonly used surgical technique during amputation is traction neurectomy, in which the nerve is transected under traction and allowed to retract more proximally to the amputated site. This could potentially lead to the formation of a neuroma contributing to RLP. Although only 4.2% of chronic PLP is associated with symptomatic neuromas, Penna et al. and de Lange et al. suggest that advanced surgical techniques seem to reduce the incidence of neuroma and PLP altogether by reconnecting the transected nerve during amputation with another healthy nerve or preserving neuromuscular matrix of the transected nerve (21, 22). In target muscle re-innervation (TMR), a transected mixed or sensory nerve was relocated to nearby or adjacent intact muscles. Then, end-to-end neurorrhaphy with a larger motor terminal end of recipient muscles was performed, reinnervating into the recipient muscles. This allowed the use of muscle contraction signals detectable by electromyography for prosthetic control. Another technique was concomitant nerve coaptation with end-to-end neurorrhaphy of the transected nerves forming a loop wrapped with a collagen nerve wrap (22). In regenerative peripheral nerve interface (RPNI), an autologous muscle graft from the amputated limb was used. The graft was wrapped around the end of the transected nerve and implanted at a location away from the surgical incision site. Of these techniques, TMR and RPNI seem to significantly reduce the incidence of PLP to 0%–56% compared to the control group's 64%–91% (23–26). Although long-term studies with substantially larger sample sizes have not been done for these surgical outcomes, these early results highlight the importance of educating surgeons to reduce the risk of PLP.

The risk factors for PLS in the post-amputation stage are the postsurgical pain, lower limb amputation with shorter residual stump, the intensities of PLS, and telescoping (9, 12, 14, 15, 19). PLP could develop quickly after the amputation; within only 7 days. Of the post-amputation patients, 85% developed PLP (27). Of note, the severity of acute post-surgical pain was found to be a weak predictor for PLP. Conversely, subacute postsurgical pain was determined to be a significant predictor for chronic PLP at 12 months (15). The lower limb amputation and more proximal amputation sites are correlated with a higher incidence of PLP (18). It is still unclear why lower limb amputations have a higher incidence rate compared to upper limb amputations. It could be speculated that maladaptive plasticity (both at the cortical levels and the lumbar spinal circuit) are potential factors. More specifically, the central pattern generators are thought to play a vital role in generating locomotion (28). Interestingly, a study by Dietrich et al. showed that the usage of leg prosthetics with a somatosensory biofeedback system reduced the severity and frequency of PLP (29). Another study by Munger et al. expressed that although the intensity of PLS significantly correlates with PLP, having phantom movement is suggested to be a protective factor against PLP (18).

In conclusion, understanding the risks associated with PLP across all stages of amputation is crucial. Interdisciplinary discussions and education are recommended to mitigate these risks effectively.
